# Cyclin-Dependent Kinase 4 and 6 Inhibitors: A Quantum Leap in the Treatment of Advanced Breast Cancers

**DOI:** 10.7759/cureus.23901

**Published:** 2022-04-06

**Authors:** Sanjana Reddy, Zineb Barkhane, Jalal Elmadi, Lakshmi Satish Kumar, Lakshmi Sree Pugalenthi, Mahlika Ahmad

**Affiliations:** 1 Obstetrics and Gynaecology, Bogomolets National Medical University, Kiev, UKR; 2 Research, Université Hassan II, Faculté de Médecine et de Pharmacie de Casablanca, Casablanca, MAR; 3 Facultad de Ciencias Médicas, Universidad Nacional Autónoma de Honduras, Tegucigalpa, HND; 4 Medicine, University of Perpetual Help System, Daisy Antonio Laperal Tamayo (DALTA), Manila, PHL; 5 Research, Ago Medical and Educational Center - Bicol Christian College of Medicine, Legazpi, PHL; 6 Research, Ziauddin University, Karachi, PAK

**Keywords:** her2-positive breast cancer, abemaciclib, ribociclib, palbociclib, cdk4/6 inhibitor

## Abstract

Breast cancer (BC) is defined as an uncontrolled growth of breast cells that affected 2.3 million women in 2020 alone. Until a few years earlier, radiotherapy and chemotherapy were the most commonly used treatments in treating BC; however, many trials and studies were conducted to test the competence of cyclin-dependent kinases 4/6 (CDK4/6) in arresting the cell cycle, and it was found that they were highly influential in halting the disease from progressing. Palbociclib, ribociclib, and abemaciclib are the three drugs that have been approved by the US Food and Drug Administration (FDA) and are even more efficient when used in combination with aromatase inhibitors and fulvestrant. This article aimed to explain the effect of CDK4/6 inhibitors on tumor cells and their efficacy in combination with other drugs. We further explored the development of resistance to these treatments and future possibilities.

## Introduction and background

Breast cancer (BC) ranks first as the most prevalent type of malignancy and second in fatality in women [1]. It is more common in women aged >45 years and rarely affects women under 40 years (4%-6%) [2]. According to a survey conducted from 2004 to 2008, the incidence rate of breast cancer was the highest among non-Hispanic women and the lowest among Asian-Americans. In contrast, the mortality rate was the highest among African-Americans, owing to a late diagnosis, and the lowest among Asian-Americans [3]. The results from the Women’s Health Initiative (WHI) trial revealed that the use of hormonal replacement therapy (HRT) in combination with estrogen and progestin for six to seven years doubled the risk of BC [4]. Alcohol consumption, family history, and radiation exposure at a young age are one of the leading risk factors that can cause BC [5]. Other main causes include BRCA1 and BRCA2 gene mutations, which result in triple-negative and estrogen receptor (ER)-positive tumors, respectively [1]. The gold standard for BC diagnosis is mammography along with clinical examination. However, the accuracy of mammograms is low in premenopausal women since they have a higher breast density; hence, screening sonography and breast MRI are also used to detect tumors [6]. Cancer cells are known to evade extracellular and intracellular signals, which often aid in limiting proliferation. The retinoblastoma (RB)-associated protein regulates cell cycle progression; however, when cyclin D collaborates with cyclin-dependent kinase 4 (CDK4) or its homolog cyclin-dependent kinase 6 (CDK6), it hyperphosphorylates RB, and its repressive impact on the E2F family of transcription factors is impaired. It could create a situation of unprecedented cell division, which cannot be regulated. The overexpression of CDK4/6 is usually seen in luminal types of breast cancer [7-9]. Since breast cancer is one of the leading causes of death, it is imperative to find a way to treat both early and advanced cancers efficiently. Palbociclib, ribociclib, and abemaciclib, three orally highly selective CDK4/6 inhibitors, were recently authorized by the US Food and Drug Administration (FDA) and the European Medicines Agency (EMA) to treat hormone receptor (HR)-positive advanced or metastatic BC [10]. The goal of this review is to accentuate the pharmacological framework, long-term consequences, efficacy of the treatment, its usage in combination with other forms of treatment, similarities, and differences between the three drugs, and the development of resistance to CDK4/6 inhibitors.

## Review

Biological rationale for CDK4/6 inhibitors in cell cycle

A decisive feature of life is its ability to reproduce, which is done through a cascade of reactions and mechanisms, commonly called the cell cycle. The cell cycle regulates the replication of deoxyribonucleic acid (DNA) and the segregation of the newly formed DNA into a new daughter cell [11]. The cells in a human body primarily exist in a quiescent state, where they do not undergo differentiation and proliferation unless a need arises. There are specific restriction points that control this decision; however, once that checkpoint is crossed, the cells move from the G1 phase to the S phase, where they start DNA replication and then proceed to G2, where the cell begins protein synthesis and prepares for mitosis [12]. Checkpoints may also be present in the S phase and G2 phase to activate DNA repair mechanisms and reevaluate if the DNA replication is complete [13]. Cancer is characterized by the dysregulation of these restriction points, which triggers an uncontrolled proliferation [7]. Progression through each phase of the cell cycle is strictly regulated by several cell cycle components, such as cyclins, CDKs, and CDK inhibitors, through phosphorylation and dephosphorylation reactions [14]. At specific cell cycle stages, different cyclins bind to different CDKs to create unique complexes that move the cell from one phase to the next [15]. Tumor suppressor RB protein is a critical negative regulator of the cell cycle that inhibits G1 transition by forming multiprotein complexes that bind to E2F factors and prevent premature cell division [16]. The CDK4/6 phosphorylates the RB protein, resulting in the release of E2F transcription factors and the activation of a transcriptional program, which activates a cascade of reactions that promotes progression into the S phase and initiates DNA replication [17,18]. Cyclin A is vital during the S phase; cyclins A and B are necessary for progression to the G2 phase and mitosis.

CDK inhibitors are a family of negative regulators that comprise two groups: inhibitors of CDK4 (INK4) and kinase inhibitor protein (KIP) [19]. The KIP family reduces the activity of the whole CDK complex, but the INK4 family indirectly inhibits the cyclin from binding by causing allosteric changes and transforming the binding site [20]. The RB comes from a family of proteins that includes a pocket (p) region that binds cellular targets [21]. pRB is in an inactivated form in G1 since it is present in a hyperphosphorylated state throughout the cell cycle, albeit dephosphorylated by mitosis [13]. The disruption of the CDK-RB pathway is one of the key elements in the pathogenesis of breast cancer [22]. In HR-positive breast tumors, estrogen stimulates the production of cyclin D1, which amplifies CDK4/6 activity, resulting in the hyperphosphorylation of RB and cell cycle promotion [23]. Estrogen signaling is vital for the tumor’s development, proliferation, and sustenance [24]. Generally, endocrine therapy (ET) is the treatment choice in metastatic ER-positive BC, but not all cancers respond to it, and the incidence of resistance is high. As a result, it has been postulated that inhibiting cell cycle regulators such as CDKs might be a valuable means of targeting ER BC [25].

Development of CDK inhibitors

CDK inhibitors are a group of pharmacological drugs that target abnormal CDK activity in cancer cells. The ATP-binding regions of proteins are targeted by all CDK4/6 inhibitor drugs [26]. The first CDK inhibitors were pan-CDK inhibitors, whose future development was hampered by difficulties such as pharmacokinetics, infeasible dose regimens, and toxicity [27,28]. In contrast, the agents recently approved by the FDA for use in breast cancer are oral highly selective inhibitors of CDK4/6 and include palbociclib, ribociclib, and abemaciclib [28].

Palbociclib

Palbociclib, sold under the brand name Ibrance, is an orally bioavailable drug created by Pfizer and approved by the FDA in February 2015 [29]. It is a CDK4/6 inhibitor with an IC50 of 9-15 nmol/L that functions by binding to the ATP pocket. It is vital to be informed that it presents with no activity against any other kinases. The CDK4/6 kinase and its coregulatory partner cyclin D assist in the G1-S transition. As a result, inhibiting this step hinders cell cycle progression in cells where the phosphorylation of RB protein and the activation of E2F transcription factors occur [30]. In preclinical investigations, it has been found that palbociclib selectively suppresses the development of ER-positive breast cancer cells, functions symbiotically with antiestrogens, and overcomes endocrine resistance [31]. These discoveries prompted the development and implementation of Palbociclib: Ongoing Trials in the Management of Breast Cancer (PALOMA). PALOMA-1 is an open-label, randomized cohort study where palbociclib plus letrozole was compared to letrozole alone as first-line treatment in 400 postmenopausal women with ER-positive, human epidermal growth factor receptor 2 (HER2)-negative advanced breast cancer. Compared to letrozole alone, the median progression-free survival (mPFS) for the combined therapy group increased by 10 months, whereas the median overall survival (mOS) was almost the same in both groups [32]. The FDA-approved palbociclib along with an aromatase inhibitor is the first line of therapy in postmenopausal women with HR-positive, HER2-negative ABC based on the success of PALOMA-2 trials, which showed an mPFS of 24 months in women treated with palbociclib and letrozole [33]. Phase III PALOMA-3 study carried out trials on the efficacy of palbociclib in combination with fulvestrant, which showed an increase in PFS when used along with fulvestrant [34,35]. TRend, a phase II trial, is analyzing the efficiency of palbociclib as monotherapy or in combination with ET in postmenopausal women with ER-positive, HER2-negative advanced breast cancer who have disease progression with previous therapy [36]. Pfizer is collaborating with the German Breast Group to supervise PENELOPE-B, a phase III trial of palbociclib in patients with ER-positive, HER2-normal, high-risk, early-stage breast cancer who still have residual disease after being treated with neoadjuvant chemotherapy and surgery [37]. A phase II research is also underway to evaluate the effectiveness of palbociclib in combination with an aromatase inhibitor in patients with HR-positive, HER2-negative, stage II/III invasive breast cancer [38].

Neutropenia, alopecia, leukopenia, and fatigue are common adverse events following the use of palbociclib [32]. Neutropenia after using palbociclib plus letrozole is different from neutropenia after chemotherapy since it is generally not linked with fever self-limiting and is characterized by recovery after a brief dose interruption or cycle delay [32]. Palbociclib can disrupt glucose homeostasis in humans in conjunction with the endocrine pancreas and effects on the eye, tooth, kidney, and adipose tissue thought to be related to the endocrine changes/glucose dysregulation.

Ribociclib

The FDA-approved ribociclib was sold under the brand name Kisqali in March 2017. It is an ATP-competitive, highly selective CDK4/6 inhibitor with a dose-dependent anticancer effect that showed excellent efficiency in treating ER-positive breast cancers and activating phosphatidylinositol 3-kinase (PIK3CA) and HER2 pathways [39]. Based on knockout animal studies, CDK4 is unnecessary for normal mammary tissue development. Still, it is essential to form Ras-induced mammary tumors, indicating a possible therapeutic window for minor hazardous therapy [40]. It reduces the association between cyclin D1 and CDK4 and cyclin D3 and CDK6 complexes. It is four times more selective to CDK4 than CDK6 [41,42]. It stops RB from being phosphorylated by CDK4/6, which blocks the release of E2F transcription factors and the production of genes involved in cell cycle progression, resulting in cell cycle arrest in the G1 phase [43-45]. The Mammary ONcology Assessment of LEE011’s Efficacy and SAfety (MONALEESA-2) conducted trials on postmenopausal HR-positive and HER2-negative advanced breast cancer women who received no previous systemic treatment in 2016. After 18 months, these trials saw a rise in PFS (63%) in women given ribociclib compared to 42.2% for patients in the placebo group. It demonstrated a decreased risk of progression by 44% after including letrozole [46]. Ribociclib therapy extended PFS regardless of PIK3CA or TP53 mutant status; total Rb, Ki-67, or p16 protein expression; or CDKN2A, CCND1, or ESR1 mRNA levels [46]. MONALEESA-3, a phase III trial, conducted investigations on 726 postmenopausal women with HR+/HER2-negative advanced breast cancer who already had one course of ET [47]. Some of them were given ribociclib plus fulvestrant. In contrast, the others were given placebo plus fulvestrant. Those in the ribociclib plus fulvestrant group had substantially longer median PFS (20.5 months versus 12.8 months) and objective response rate (ORR) than patients in the placebo plus fulvestrant arm [47]. MONALEESA-7 investigated 672 premenopausal women who were not previously treated with ET and were randomly given ribociclib or placebo in combination with tamoxifen or nonsteroidal aromatase inhibitor therapy as regards ovarian function suppression (OFS) with goserelin. Their group had a PFS of 23.8 months, which was comparatively higher than the group that received a placebo [48]. Moreover, independent of ET, individuals treated with ribociclib had a higher mean change in pain score and a substantially longer median time to deterioration than patients in the placebo group [48]. It is the only CDK4/6 inhibitor licensed with fulvestrant as a first- or second-line therapy for postmenopausal women with HR+/HER-positive advanced breast cancer [49]. Many ongoing trials explore ribociclib as the first line of treatment in patients with HR+ early and advanced disease. Interestingly, two of these studies are evaluating ribociclib potency in patients who have progressed on a prior CDK4/6 inhibitor: (1) phase II MAINTAIN trial, in which patients receive either ribociclib plus fulvestrant or placebo plus fulvestrant, and (2) TRINITI-1 trial, which is exploring the triplet combination of everolimus plus exemestane plus ribociclib [40]. Over three years, the cost savings of ribociclib in association with an aromatase inhibitor in postmenopausal women were evaluated. Direct and indirect expenses were examined, and a total savings of more than US$3 million per patient for the US payer formulary were demonstrated, showing that ribociclib may be employed as a cost-effective treatment choice [50].

Palbociclib and ribociclib both have similar toxicity profiles. Febrile neutropenia and alopecia are typical, whereas elevated aminotransferases and prolonged QT intervals are also seen in ribociclib toxicity [51].

Abemaciclib

The FDA-approved abemaciclib was sold under the brand name Verzenio in September 2017. The RB tumor suppressor protein-mediated pathway regulates passage past the G1 restriction point (R). The interaction of CDK4 and CDK6 with D-type cyclins is required to activate the RB-mediated pathway, which leads to the creation of active CDK4/CDK6 and the subsequent phosphorylation of RB. The CDK4/6 phosphorylation of RB and other proteins also promotes the transcription of genes involved in cell cycle-independent functions such as signal transmission, DNA repair transcriptional regulation, and mRNA processing [52]. It has greater selectivity for CDK4 than CDK6, and its effects on brain metastases are being investigated since it crosses the blood-brain barrier [53]. MONARCH 1, a phase II trial, studied 132 patients who had failed two ETs and one or two chemotherapies; the mPFS was six months, and the mOS was 17.7 months [54]. MONARCH 2 was a phase III study of women whose advanced breast cancer had progressed during the previous ET. Patients were ambiguously allocated to receive abemaciclib plus fulvestrant or placebo plus fulvestrant. The results showed that the use of abemaciclib raised the PFS as compared to the use of placebo (16.4 months versus 9.3 months) and mOS (46.7 months versus 37.3 months) remarkably [55]. MONARCH 3 has a similar setting as PALOMA-2 and MONALEESA-2, which employed letrozole in combination with and without abemaciclib. The PFS significantly increased (28.2 months versus 14.7 months) with abemaciclib. Hence, the FDA approved it in 2018 to be used as the first line of treatment in combination with an aromatase inhibitor. The toxicity profile is notably different from the other two, as abemaciclib has a lower incidence rate of neutropenia but a higher rate of diarrhea. Deep vein thrombosis (DVT) and pulmonary embolism were also seen in a small fraction of patients [56].

Both palbociclib and ribociclib are given intermittently (21 days on, seven days off), whereas abemaciclib is given continuously [49].

Table 1 shows the details of the studies on palbociclib, ribociclib, and abemaciclib and in combination with other treatments.

**Table 1:** Population data table mPFS: median progression-free survival, mOS: median overall survival, ORR: objective response rate

References	Phase	Method	Sample population	Design	Outcomes
Finn et al. (PALOMA-1) [32]	II	Cohort study	400 postmenopausal women	Control group I: palbociclib plus letrozole; control group II: only letrozole	mPFS: group I, 20.2 months; group II, 10.2 months; mOS: group I, 37.5 months; group II, 33.3 months; ORR: group I, 36%; group II, 27%
Finn et al. (PALOMA-2) [33]	III	Double-blind study	666 postmenopausal women who have never received any form of treatment for their disease	Control group I: palbociclib plus letrozole; control group II: placebo plus letrozole	mPFS: group I, 24.8 months; group II, 14.5 months; ORR: group I, 42.1%; group II, 34.7%
Cristofanilli et al. (PALOMA-3) [34,35]	III	Double-blind study	521 women with disease that relapsed or progressed during ET	Control group I: palbociclib plus fulvestrant; control group II: placebo plus fulvestrant	mPFS: group I, 9.5 months; group II, 4.6 months; ORR: group I, 10.4%; group II, 6.3%
Hortobagyi et al. (MONALEESA-2) [46]	III	Placebo-controlled study	668 postmenopausal women who have not received any previous systemic therapy	Control group I: ribociclib plus letrozole; control group II: placebo plus letrozole	mPFS: group I, not reached; group II, 14.7 months; ORR: group I, 40.7%; group II, 27.5%
Tripathy et al. (MONALEESA-7) [48]	III	Double-blind, placebo-controlled study	672 pre- and perimenopausal women with no previous ET	Control group I: ribociclib plus goserelin plus tamoxifen/letrozole; control group II: placebo plus goserelin plus tamoxifen/letrozole	mPFS: group I, 23.8 months; group II: 13 months; mOS: group I, not reached; group II, 40.9 months
Sledge Jr. et al. (MONARCH 2) [55]	III	Double-blind study	669 women whose disease has progressed during the previous ET	Control group I: abemaciclib plus fulvestrant; control group II: placebo plus fulvestrant	mPFS: group I, 16.4 months; group II, 2.3 months; ORR: group I, 48.1%; group II, 21.3%
Goetz et al. (MONARCH 3) [56]	III	Double-blind, cohort study	493 postmenopausal women with no previous systemic treatment for their disease	Control group I: abemaciclib plus anastrozole/letrozole; control group II: placebo plus anastrozole/letrozole	mPFS: group I, not reached; group II, 14.7 months; mOS: group I, not reported; group II, not reported

How are they better than the standard treatment?

Endocrine therapy is the first line of treatment for breast cancers, but many do not respond to it. In this case, chemotherapy is indicated, but this mode of therapy has a detrimental effect on healthy cells. Third-generation CDK inhibitors may selectively inhibit CDK4/6 and control the cell cycle by reducing the G1 to S phase transition, demonstrating an ideal balance between anticancer activity and general toxicity to healthy cells [57]. After analyzing the phase III PEARL study results, it was deduced that there was no change in the time of progression-free survival in patients treated with CDK4/6 inhibitors and endocrine therapy and patients who were given chemotherapy. Still, there was an improvement in toxicity profile and reduced time to deterioration of global health status. In addition, combination therapy appeared to improve the quality of life and tolerability [58].

FDA precautions and guidelines for patients receiving the approved CDK4/6 inhibitors in treating breast cancer are given in Table 2 [59].

**Table 2:** FDA precautions and guidelines for patients receiving the approved CDK4/6 inhibitors AI: aromatase inhibitor

	Palbociclib	Ribociclib	Abemaciclib
Brand name	Ibrance	Kisqali	Verzenio
Starting dose	125 mg	600 mg	150 mg with AI/fulvestrant, 200 mg as monotherapy
Dosing frequency	Once daily	Once daily	Twice daily
Treatment period	21 days	21 days	28 days
Rest period	Seven days	Seven days	No need to rest
CBC monitoring	Once every two weeks during the first two cycles and then only at the start in the next four	Once every two weeks during the first two cycles and then only at the start in the next four	Once every two weeks for the first two months and monthly for the next two months
EKG monitoring	Not necessary	On day 14 of cycle 1 and day 1 of cycle 2; monitor electrolytes at the start of each cycle	Not required
LFT monitoring	Not necessary	Once every two weeks during the first two cycles and then only at the start in the next four	Once every two weeks for the first two months and monthly for the next two months
Fetal toxicity	Patients should be well informed of the potential risks to the fetus and advised to use contraception	Patients should be well informed of the potential risks to the fetus and advised to use contraception	Patients should be well informed of the potential risks to the fetus and advised to use contraception
Diarrhea advice	Not necessary	Not necessary	Counsel patients to start antidiarrheal therapy in the case of loose stools and increase fluid intake
VTE advice	Not necessary	Not necessary	Monitor patients for any signs of thrombosis or pulmonary embolism

Combination with other treatments

CDK4/6 inhibitors do not allow the CDK4/6 to phosphorylate RB, which enables it to suppress the E2F transcription factor family, which results in the decreased transcription of proliferation proteins. The decreased transcription of DNA methyltransferase 1 (DNMT1) protein results in the increased efficiency of tumor cell antigen synthesis and the inhibition of regulatory T-cell proliferation [60]. CDK6 inhibition increases the nuclear factor of activated T-cell (NFAT) proteins in the nucleus, amplifying effector T-cell activity [61]. These findings suggest a possible role in combining treatment with CDK4/6 inhibitors and immunotherapy. Most of the combinations entail the PI3K pathway; the TRINITI-1 trials explored triplet therapy, exemestane plus ribociclib plus everolimus, after disease progression while using CDK4/6 and ET therapy. The ORR and PFS were revealed to be less than that seen in the BOLERO-2 trial, which involved exemestane plus everolimus (ORR: 8% versus 9.5%; PFS: 5.7 months versus 6.9 months) [62,63]. This disparity might be attributed to the patients’ varying endocrine sensitivity and the prevalence of PIK3CA and SR1 mutations found in 30% of TRINITI-1 trial participants [63].

Development of resistance

There are two types of CDK4/6 inhibitors: cell cycle alterations and PI3K/mitogen-activated protein kinase one signaling [22]. RB1 deficiency is linked to new CDK4/6 inhibitor resistance, and the reduction of RB1 expression in hormone receptor-positive breast cancer cells over time in the presence of palbociclib has been documented [64]. Apart from RB deficiency, through CDK2 activation, cyclin E1 and cyclin E2 ectopic overexpression can result in a bypass track and enhance resistance to antiestrogen treatments, including palbociclib monotherapy in vitro [65]. The PALOMA-3 cohort trials revealed that the effect of palbociclib was reduced in patients with higher levels of cyclin E1 (the mPFS in higher cyclin E1 versus lower cyclin E1 is 7.6 months versus 14.1 months ). CDK6 overexpression resulted in lower estrogen receptor expression and, consequently, resistance to antiestrogen drugs, indicating that resistance to the CDK4/6 inhibitor and the ET partner arises through this mechanism [66].

Some investigations of tumor specimens resistant to CDK4/6 inhibitors revealed various possible resistance pathways, including upstream changes in AKT1, KRAS, HRAS, NRAS, FGFR2, and ERBB2 [66]. As a putative resistance mechanism, inactivating mutations in the FAT atypical cadherin 1 (FAT1) gene have been discovered, with its loss leading to the activation of YAP1 via the Hippo pathway and consequent increase in CDK6 expression [67]. Of the patients with ER-positive BC, 40% were found to have a mutation in the PIK3CA catalytic subunit [9]. The PI3K/mTOR pathway has been revealed to be increased in response to persistent exposure to CDK4/6 inhibitors, which in turn upregulates cyclin D. This active cyclin D can activate CDK2, which promotes cell cycle progression even in the absence of CDK4 and CDK6 [64]. The PI3K-PDK1 signaling pathway has been linked to CDK4/6 inhibitor resistance, with ribociclib-resistant BC cell lines exhibiting an increase in PDK1 levels after drug exposure, culminating in AKT pathway activation [68]. Acquired resistance to CDK4/6 inhibitors is a nearly universal certainty, which has sparked considerable interest in investigating probable reasons for resistance strategies for overcoming it and recognizing it [69].

Future outlook

Few ongoing trials are looking at using CDK4/6 inhibitor in HR+/HER2+ metastatic BC [70]. A CLEOPATRA-like routine is being tested, stopping the chemotherapy and going on with trastuzumab and pertuzumab and later including ET for HR+/HER2+ advanced breast cancer [71]. PATINA, a randomized phase III trial, analyzes the effectiveness and safety of palbociclib along with ET- and HER2-targeted therapy after the initial treatment [72]. CDK4/6 inhibitor is also being studied for its efficacy and safety in early-stage breast cancer in neoadjuvant, adjuvant, and post-neoadjuvant settings. The phase II neoadjuvant NeoPalAna trial found that combining palbociclib with anastrozole improves cell cycle arrest [73]. Preclinical and clinical evidence suggests that CDK4/6 inhibitors might be utilized alone or in combination with other chemotherapy or targeted treatments in various tumor situations [74]. CDK4/6 inhibitor as a single drug in ovarian cancer patients does not appear to be promising and is unlikely to be pursued further unless specific biomarkers of action are established. Based on the biology of germ cell tumors (GCT), they typically overexpress cyclin D2; it may be worthwhile to investigate if the CDK4/6 inhibitor is particularly active in these histotypes. A phase II trial that included patients with recurrent ovarian cancer were tested with ribociclib and letrozole. Patients with low-grade tumors showed high response rates, which prompted this treatment to be explored thoroughly to treat ovarian cancers [74]. Mutations in the tumor suppressor genes TP53 are most likely found in high-grade serous ovarian cancers, and they are said to be highly sensitive to ataxia-telangiectasia mutated and Rad3-related (ATR) inhibition, making it worth testing the relation between CDK4/6 inhibitor and cisplatin, using CDKi as maintenance therapy [74,75]. There is presently no mature data to support immunotherapy in ovarian cancer patients. Considering that CDK4/6 inhibitors have a significant influence on the immune response to various cancer types, it would be intriguing to see if using CDK4/6 inhibitors could increase the efficacy of immune checkpoint inhibitors. It would change people’s lives with platinum-resistant subtypes since they have very limited therapeutic options [76].

Limitations

The main drawback of this review is that this treatment for advanced breast cancers has been approved very recently; hence, we could not review all the data as many clinical trials are still underway. Furthermore, this article does not discuss the methods of overcoming drug resistance.

## Conclusions

It is evident from the review article that breast cancer is one of the leading causes of death, and there are very few treatments that are effective in curing advanced stages of this disease. To summarize, the clinical significance of this article is to focus on the efficacy of CDK4/6 inhibitors in prolonging the life span of patients by inhibiting the growth and proliferation of cancer cells. We trust that this article can open up a new approach to treating advanced cancers by highlighting the drug’s pharmacological effect on the body and the cancer cells compared to other modes of treatment such as chemotherapy and radiotherapy. We spoke about the development of CDK4/6 inhibitors, their usage in combination with other medicines, the challenges faced due to drug resistance, and future implications. Among the three drugs, palbociclib was found to have the least number of side effects; ribociclib was found to cause arrhythmias and an increase in aminotransferases. All of them had similar efficiency in treating breast cancer. Finally, we believe that an active study through further clinical investigations and systematic analysis is essential to get a clear picture of their use in cancers.
